# Supplementary material for: The Frontal Aslant Tract and Supplementary Motor Area Syndrome: Moving towards a Connectomic Initiation Axis
Source: Cancers (Basel). 2021 Mar 5;13(5):1116. doi: 10.3390/cancers13051116 (PMC7961364; doi:10.3390/cancers13051116)
Supplement: Supplementary file 1 [file cancers-13-01116-s001.pdf]

Supplementary Material: The Frontal Aslant Tract and Supplementary Motor Area Syndrome: Moving towards a Connectomic Initiation Axis:

Robert Briggs [1], Parker G. Allan [1], Anujan Poologaindran [2,3], Nicholas B. Dadario [4,5] , Isabella M. Young [4], Syed A. Ahsan [4], Charles Teo [4], Michael E Sughrue [4,*]

[1] Department of Neurosurgery, University of Oklahoma Health Sciences Center, Oklahoma City, OK, USA
[2] Brain Mapping Unit, Department of Psychiatry, University of Cambridge, Cambridge, UK
[3] The Alan Turing Institute, British Library, London, UK
[4] Department of Neurosurgery, Prince of Wales Private Hospital, Sydney, Australia
[5] Rutgers Robert Wood Johnson Medical School, New Brunswick, NJ, USA

* Correspondence: sughruevs@gmail.com

Supplementary Material

Results

Network Components of the "Command and Control" Axis

The Human Connectome Project (HCP) recently published their scheme for parcellating the human neocortex based on physical and functional MRI characteristics of the brain. We have utilized this scheme to describe brain connectivity in ways which can be compared, reproduced, and utilized by neurosurgeons. The following sections detail our work related to the anatomy and connectivity of three large-scale functional networks using the HCP parcellation format. These results are based on the resting-state activity maps of relevant HCP parcellations combined with diffusion spectrum tractography to build the best possible anatomic models for these networks to date. While there is significant individual variability in the human cortex, and while gliomas are known to cause functional re-organization within the brain, these models serve as an initial starting point in discussing the structural organization of cerebral networks in the brain.

<u>The Default Mode Network</u>

The default mode network (DMN) is classically a three-part system consisting of parts of the anterior cingulate and medial frontal cortices, the posterior cingulate cortex, and the inferior parietal lobule.[8] Careful study shows this is not entirely adequate, as parts of the middle temporal gyrus, thalamus, and hippocampus strongly correlate with these regions in many datasets Figure S1 shows our model of the DMN. The anterior frontal regions include areas a24, s32, p32, and 10r; the posterior cingulate areas include areas 31a, 31pd, 31pv, d23ab, v23ab, 7m, POS1, POS2 and the retrosplenial cortex (RSC); and the inferior parietal regions include PFm, PGs, and PGi. The anterior frontal and posterior cingulate areas are clearly joined by the cingulum. We have found no evidence of a direct white matter connection between the lateral parietal regions and the medial cingulate regions, suggesting that the network is functionally constructed by the thalamocortical rhythms or by dynamic interplay with other neighboring areas.

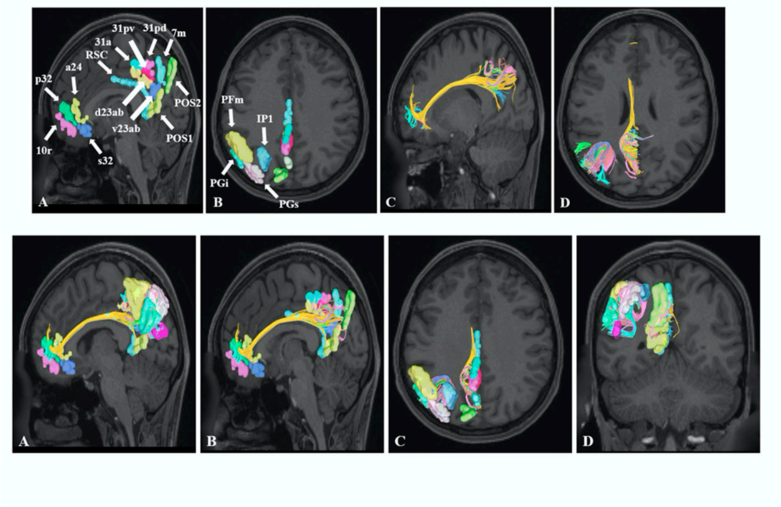

Figure S1. This figure shows our model of the DMN. The anterior frontal regions include areas a24, s32, p32, and 10r; the posterior cingulate areas include areas 31a, 31pd, 31pv, d23ab, v23ab, 7m, POS1, POS2 and the retrosplenial cortex (RSC); and the inferior parietal regions include PFm, PGs, and PGi. The first two diagrams are the key parcellations involved in the DMN, the others elucidate the tractography between these areas.

<u>The Central Executive Network</u>

The central executive network, which is also referred to as the control network, is fundamentally a fronto-polar and parietal network (Figure S2). Our model of the control network currently includes prefrontal areas 9a, 9p, 9–46d, a9-46v, and area 46; polar areas a10p and p10p; inferior frontal sulcus areas IFSa and IFSp; parietal areas PFt and PF; opercular area OP4; and intraparietal sulcus areas AIP, IP1, and IP2. These frontal and parietal areas are connected by the superior longitudinal fasciculus.

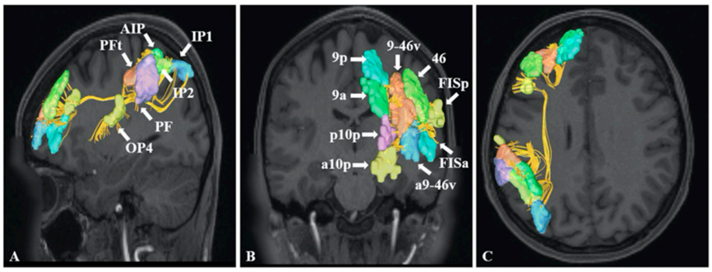

Figure S2 Parcellations of the CEN system and the relevant tractography.

<u>The Salience Network</u>

The salience network (Figure S3) is a network comprising parts of the middle cingulate cortex and anterior insula. Our model of the salience network includes cingulate areas a32pr, p32pr, and the supplementary and cingulate eye field area (SCEF). The insular regions comprising this network include the anterior ventral insula (AVI), the middle insula (MI), and frontal opercular areas FOP4 and FOP5. These regions are connected via the FAT. Notably, SCEF has also been identified as part of the SMA complex, providing a critical link between the salience network and the motor planning system.

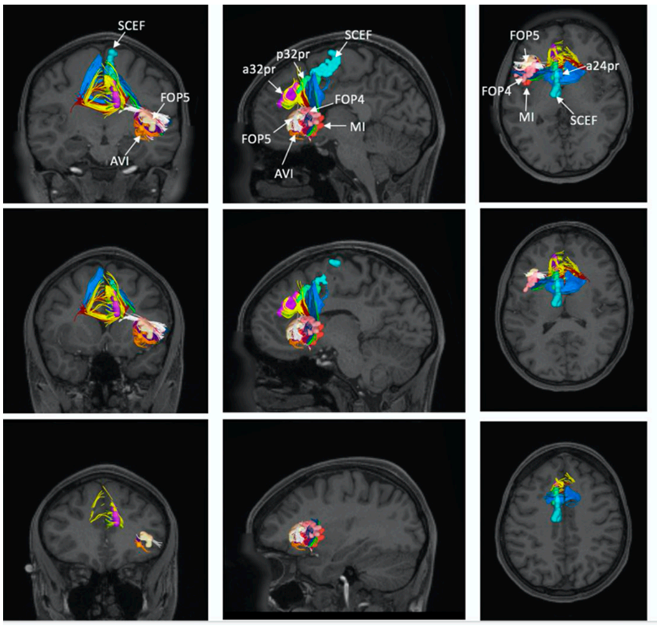

Figure S3 Parcellations of the SN system and the relevant tractography.

Parcellations of the Supplementary Motor Area

The parcellations in the medial bank of the posterior SFG include areas 6ma, 6mp, SCEF and the superior frontal language area (SFL) (Figure S4a). In addition to connecting to their immediate neighbors via u-shaped fibers, the SMA areas also connect to their contralateral analogues via the corpus callosum, the ipsilateral premotor, opercular and insular cortices via the FAT, the contralateral premotor areas via the crossed FAT, and demonstrate fibers running within the corticospinal tract (Figure S4b). Direct connections to the primary motor cortex are sparse.

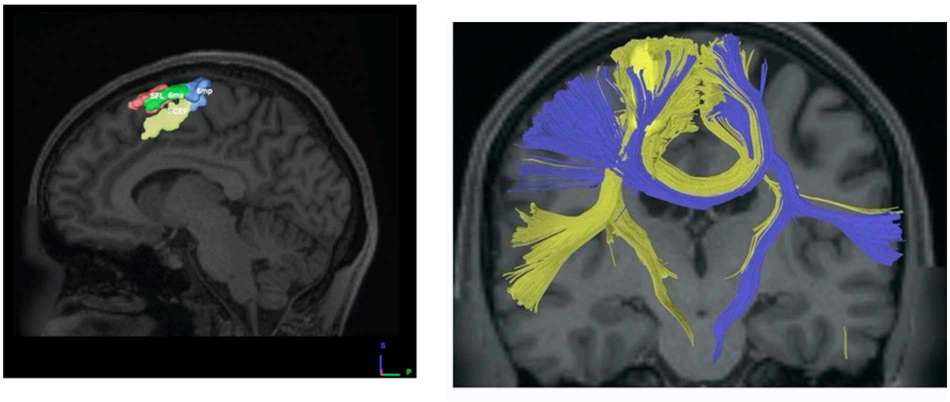

(**a**) (**b**)

Figure S4 (a) Demonstrates the key parcellations of the supplementary motor area; (b) illustrates how the SMA areas connect to the neighbors via u-shaped fibers, to their contralateral analogues via the corpus callosum and the ipsilateral premotor, opercular and insular cortices via the FAT.

Figure S5 provides a simple schematic of DMN-CEN interconnections. The SN is a third network which appears to be key in this transition. Failure to alternate or transition between these networks has been observed in minimally conscious and vegetative patients. In addition, this transitional process has been shown to be impaired in patients with schizophrenia with severe negative symptoms. Thus, it seems reasonable to hypothesize that disruption of these systems could impair a patient's ability to organize their thoughts, generate an action plan, and transition towards its execution.

Figure S6a and S6b demonstrate combined images showing the parts of the initiation system in both medial and lateral views. Several important structural aspects of these networks can be observed when they are viewed in combination. First, the networks meet in several places. One notable point is the adjacency of the CEN and DMN networks in the lateral parietal lobe. Another notable point is the adjacency of the SN and ventral premotor network, which both share connections within the FAT. Another notable relationship can be seen in the medial frontal lobe, where parts of the DMN, SN, and SMA form a lazy-S type complex straddling the anteromedial SFG, anterior cingulate, and canonical SMA regions in the form of an "initiation strip". In fact, the SMA and SN share one parcellation, SCEF. While our initial explanation of the benefit of cingulate preservation in avoiding abulia during butterfly glioma surgery focused on the DMN, this complex of adjacent areas provides a plausible model of how one escapes from internal thoughts and initiates goal-directed behavior.

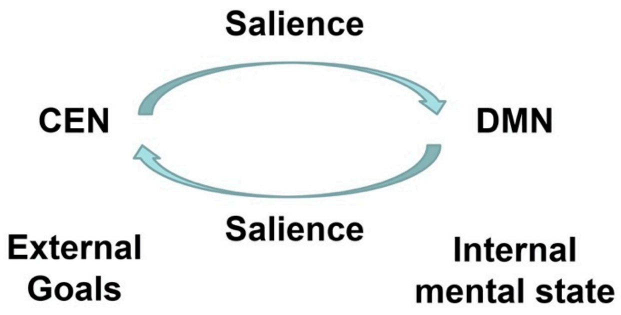

Figure S5- A simple schematic showing how the salience network mediates the transitions between the use of the DMN and the CEN systems depending on whether a person is involved in a task.

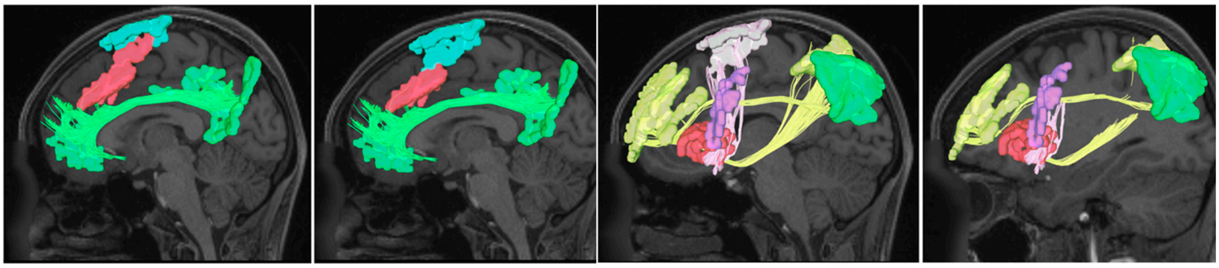

(**a**) (**b**)

Figure S6- (a) and (b) demonstrate combined images showing the parts of the initiation system in both medial and lateral views
